# Protective or Pressuring? Multi-Group Structural Path Analysis of Family–School Support and Mental Health Among Postgraduates

**DOI:** 10.3390/ejihpe15110227

**Published:** 2025-11-05

**Authors:** Ying Zhou, Jinbo Hou, Chenling Liu, Chunyan Zhou, Jingjing Song, Lin Li

**Affiliations:** Department of Psychology, Institute of Education, China University of Geosciences, Wuhan 430074, China; zhouying@cug.edu.cn (Y.Z.); houjinbo@cug.edu.cn (J.H.); liuchl@cug.edu.cn (C.L.); zhouchunyan@cug.edu.cn (C.Z.); songjingjing925@cug.edu.cn (J.S.)

**Keywords:** family function, supervisor–postgraduate relationship, mental health, self-efficacy in research, multi-group structural path analysis

## Abstract

(1) Background: With the continuous expansion of graduate education, the mental health of postgraduates has become a growing concern for both academia and society. Understanding how family and institutional resources influence psychological outcomes is critical for developing effective support strategies; (2) Methods: A cross-sectional survey was conducted among 3998 postgraduate students across China, including 3393 master’s students (51.78% female, *M* = 24.21, *SD* = 1.521) and 605 doctoral students (37.19% female, *M* = 27.77, *SD* = 2.841). Multi-group structural equation modeling was employed to examine how family functioning and supervisor–postgraduate relationships influenced mental health, research self-efficacy, and suicidal tendencies; (3) Results: The findings showed that although most structural relationships were consistent across groups, two critical pathways were nonsignificant at the doctoral stage, providing evidence of partial structural invariance; (4) Conclusions: The study suggests that while family and school support generally play a protective role, their influence varies across educational stages. Tailoring psychological interventions to the distinct needs of master’s and doctoral students is essential, offering both theoretical insights into the dual role of contextual resources and practical guidance for targeted mental health support in graduate education.

## 1. Introduction

### 1.1. Background

With the rapid global expansion of higher education, postgraduates have become a vital force driving knowledge production and innovation. However, as universities raise academic standards and impose publication requirements, academic research pressures have intensified. This has resulted in widespread psychological distress among postgraduates, with the prevalence of depression and anxiety reported to be six times higher than that of the general population (Evans et al., 2017). More concerning are the increasingly frequent cases of doctoral students who die by suicide globally, which not only result in irreversible talent loss but also expose systematic shortcomings in mental health support within the graduate education system (Guidotti et al., 2024; Meda et al., 2021).

Although numerous studies have examined anxiety and depression among postgraduates, prevalence rates vary widely, from 9.2% to 86.0%, due to methodological differences and variations in symptom presentation (P. Wang et al., 2018; Garcia-Williams et al., 2014). These inconsistencies emphasize the need to understand the contextual factors shaping postgraduates’ mental health, particularly their family and institutional environments.

### 1.2. Theoretical Support

#### 1.2.1. Life Course Perspective and the “Thick-Skinned Bias”

According to life course theory, doctoral students are in a period of “emerging adulthood,” facing multiple social expectations related to career, family, and financial independence. These overlapping pressures shape how they perceive and respond to stress. The prevailing societal notion of a “thick-skinned bias”, the assumption that individuals develop greater resilience through academic progression, remains unverified. In fact, empirical research shows that repeated stress exposure often increases vulnerability rather than resilience (Haim-Nachum et al., 2022; Layne et al., 2008). Levecque et al. (2017) found that 32% of doctoral students were at risk of common mental disorders, driven primarily by workload, low job control, and work–family conflict. Similarly, Thomas Tobin et al. (2021) and Miller et al. (2019) demonstrated that disadvantaged groups accumulate greater stress and fewer coping resources, contradicting adaptation-level theory (Helson, 1964). In China, traditional cultural values emphasizing filial duty and social success intensify these burdens. The advisor-responsibility system further amplifies supervisory pressure, making the quality of the supervisor–postgraduate relationship a decisive factor in mental health outcomes.

#### 1.2.2. Social Support as Buffer and Burden

Social support theory (Cohen & Wills, 1985) proposes that emotional, informational, and practical support from significant others can buffer stress, especially under high-pressure conditions. However, support is not uniformly beneficial, and it may also function as a burden by heightening expectations or role conflict (Dolson & Deemer, 2022; Levecque et al., 2017). Experimental evidence shows that even brief social-belonging interventions can significantly improve academic performance and well-being (Walton & Cohen, 2011). These findings underscore the importance of precisely targeted support systems. Yet, many studies treat postgraduates as a homogeneous group, neglecting developmental distinctions. Arnett (2000) noted that individuals in emerging adulthood, typical of master’s students, focus on exploration, whereas doctoral students emphasize stability and social roles. Failing to account for these differences may obscure crucial variations in coping mechanisms and mental health outcomes.

#### 1.2.3. Research Gaps and Study Objectives

Previous research often isolates either family function or supervisory relationships, rarely examining their joint effects (DeWit et al., 2016; Aass et al., 2022). Furthermore, few studies explore the dual nature of these supports as both protective and pressuring forces. In China’s collectivist and hierarchical educational culture, this issue becomes even more salient, as family expectations and mentor authority intersect to shape students’ psychological adaptation. To address these gaps, this study constructs a multi-group structural equation model (SEM) integrating family function and the supervisor–postgraduate relationship as parallel sources of support. By comparing master’s and doctoral students, we aim to clarify (1) how home and school environments influence mental health, self-efficacy in research, and suicidal ideation, and (2) whether these pathways differ across educational stages. This approach enriches existing models by revealing the complex balance between support and strain in postgraduate education.

### 1.3. Research Hypotheses

As graduate education expands, postgraduates (especially doctoral candidates) face unprecedented psychological and academic challenges (Evans et al., 2017; Levecque et al., 2017). Family and institutional support systems have thus become key determinants of mental health and research adaptation. Based on prior theories and evidence, we propose the following hypotheses.

#### 1.3.1. The Relationship Between Home and School Environment and Mental Health

Ample evidence demonstrates that the home and school environment significantly shapes postgraduates’ mental health and adaptation. Family function reduces anxiety and enhances well-being (Chen et al., 2023), while positive supervisor–postgraduate relationships foster belonging and academic engagement (Rose, 2005). Mentor support also predicts higher research self-efficacy and motivation (Lev et al., 2010). However, the mediating role of research pressure remains contested: some studies suggest it directly triggers emotional problems, whereas others highlight indirect cognitive or motivational pathways (C. Liu et al., 2019; Farsani & Ghorbani, 2024).

At higher academic stages, suicidal ideation mechanisms are particularly complex. While psychological distress is a direct predictor (Levecque et al., 2017), whether home–school environments mitigate such risks through a chain mediation process remains unclear. According to social support theory, emotional, informational, and instrumental support from family members and mentors, buffers stress and promotes well-being (Cohen & Wills, 1985). For postgraduates, family function entails emotional connectedness, communication, and adaptability, whereas the supervisor–postgraduate relationship embodies trust, guidance, and empathy. Empirical studies show that supportive family and school dynamics enhance emotional stability and belonging, fostering resilience during research stress (Walton & Cohen, 2011). Effective communication and joint decision-making within families reduce anxiety and promote well-being (Barzoki & Toikko, 2025; Berryhill & Smith, 2021; Xu et al., 2021). Conversely, strained family ties, excessive expectations, or communication breakdowns heighten risks of depression and suicidal ideation (Q. Yang et al., 2022). In Chinese culture, where filial responsibility and family honor are emphasized, these pressures may intensify internalized stress (J. Zhang & Goodson, 2011).

Similarly, the supervisor–postgraduate relationship exerts a profound influence on mental health. Supportive mentorship enhances confidence and identity (Rose, 2005), and in Chinese samples, closer supervisor–postgraduate relationships correlate with lower anxiety (Le et al., 2021). However, when supervision becomes distant, controlling, or instrumental, it can induce oppression and even “academic trauma” (Brown et al., 2020). High parental or supervisory expectations without emotional understanding may also trigger role conflict, loneliness, and self-doubt (Wentzel et al., 2016).

#### 1.3.2. The Influence of Home and School Environment on Self-Efficacy in Research

Self-efficacy in research, defined as confidence in one’s ability to conduct research tasks (Bandura, 1997), is a critical mediating variable in academic adaptation. Families with higher educational literacy and emotional support foster stronger research motivation (Eccles & Wigfield, 2002), while supportive mentorship enhances persistence and confidence (Paglis et al., 2006). In contrast, students from economically disadvantaged or culturally under-resourced families may experience “hidden stress” and “resource mismatch,” leading to low confidence and unclear career paths (Chang et al., 2020). Low-quality supervision characterized by poor feedback and limited communication undermines self-efficacy and increases burnout risk (Stubb et al., 2011). Under the influence of Chinese “familial self-continuity” culture, postgraduates often face dual expectations, meeting family aspirations while pursuing academic excellence, which can transform supportive relationships into stressors. This study thus hypothesizes that self-efficacy mediates the relationship between the home and school environment and mental health and suicidal ideation, with differential mechanisms across educational stages.

## 2. Methodology

### 2.1. Participants

This study employed a cross-sectional design and collected data from 3998 postgraduates from universities in China. Among them, 3393 were master’s students (*M*_age_ = 24.21, *SD* = 1.52) and 605 were doctoral students (*M*_age_ = 27.77, *SD* = 2.84).

### 2.2. Measures

#### 2.2.1. Home and School Environment

Family function was assessed using the General Family Function Scale developed by Zou et al. (2010), which has demonstrated good reliability and validity. The scale consists of six items rated on a 5-point Likert scale (1 = “strongly disagree” to 5 = “strongly agree”), with total scores ranging from 6 to 30. Higher scores indicate better overall family function. The internal consistency was high (*Cronbach’s α* = 0.947) and the content validity index was 0.889.

The quality of the supervisor–postgraduate relationship was measured using the Leader–Member Exchange (LMX) scale by Graen and Uhl-Bien (1995), revised for graduate supervisor–postgraduate relationships by Yu Xiaomin and colleagues. This scale includes seven items rated on a 5-point Likert scale, where higher scores represent better perceived relationship quality. An example item is, “I trust my advisor and support all of their research-related plans and decisions.” The scale is reliable (*Cronbach’s α* = 0.835) and content validity index was 0.969.

#### 2.2.2. Academic Research Pressure

Academic research pressure was measured using a three-item unidimensional scale developed by F. Zhou (2009), assessing individuals’ cognitive and emotional reactions to academic tasks (e.g., “I feel anxious about how to complete research tasks”). Responses are rated on a 6-point Likert scale (1 = “strongly disagree” to 6 = “strongly agree”), with higher scores indicating greater perceived academic research pressure (*Cronbach’s α* = 0.894) and the content validity index was 0.717.

#### 2.2.3. Mental Health

Mental health was assessed using the Symptom Checklist-90 (SCL-90) developed by Z. Y. Wang (1984), which contains 90 items and 10 subscales, including somatization, obsessive–compulsive symptoms, interpersonal sensitivity, depression, anxiety, hostility, phobic anxiety, paranoia, and psychoticism, as well as other factors such as sleep and diet. Respondents rated their experiences over the past week on a 5-point scale (1 = “not at all” to 5 = “extremely”). Higher subscale scores indicate more severe symptoms. Symptom levels are categorized as moderate (within ±1 SD), elevated (±2 SD), or high/low (beyond ±2 SD). The scale’s internal consistency was excellent (*Cronbach’s α* = 0.981), and the content validity index was 0.988.

#### 2.2.4. Self-Efficacy in Research

Self-efficacy in research was measured using a scale developed by Y. J. Zhang et al. (2013), which includes three items, such as “I am confident in my ability to conduct research” and “I am capable of handling difficulties in research.” The total score ranges from 3 to 15, with higher scores reflecting greater self-efficacy. The scale’s reliability was *Cronbach’s α* = 0.953 and the content validity index was 0.858.

#### 2.2.5. Suicidal Ideation and Behavior

Suicidal ideation and behavior were assessed using the Suicide Behavior Screening Questionnaire (SBSQ) developed by L. Yang et al. (2021), which consists of eight items. Items 1–2 screen for suicidal thoughts, 3–5 for past attempts, and 6–8 for actual attempts. The scale uses an 8-point Likert format, with higher scores indicating greater suicide risk. Participants were categorized as (1) those with ideation but no attempts, (2) those with past attempts and ideation, or (3) those with actual attempts and ideation. The scale demonstrated acceptable reliability (*Cronbach’s α* = 0.839), and the content validity index was 0.774.

### 2.3. Data Analysis

Since some variables (e.g., suicidal tendency) were not normally distributed, we used Maximum Likelihood Estimation with robust standard errors (MLR) in SEM to accommodate non-normality, which does not require strict normality assumptions (Falk, 2018).

Data were analyzed using SPSS 27.0 and Amos 28.0. Structural Equation Modeling (SEM) with Maximum Likelihood Estimation was employed to test the hypothesized model, including home and school environment (family function and supervisor–postgraduate relationship), academic research pressure, SCL-90, self-efficacy in research, and suicidal tendency. Multi-group analysis was used to compare model differences between master’s and doctoral students. Model fit was evaluated using CFI (>0.90), RMSEA (<0.08), and SRMR (<0.06) criteria (Kline, 2015). Mediation effects were tested using 5000 bootstrap samples and 95% bias-corrected confidence intervals; mediation was deemed significant if the interval excluded zero (Preacher & Hayes, 2004). Measurement invariance across groups was assessed using ΔCFI < 0.01 and ΔRMSEA < 0.015, followed by partial constraint release to explore group differences. Gender and discipline were included as covariates, and missing data were handled using full information maximum likelihood estimation (Acock, 2005).

### 2.4. Common Method Bias and Correlational Analysis

Harman’s single-factor test was conducted to examine common method variance. Twenty-four factors had eigenvalues greater than 1. The first factor accounted for 25.30% of the variance, which is well below the 40% threshold, indicating that common method bias was not a significant concern (H. Zhou & Long, 2004).

## 3. Results

### 3.1. Descriptive Statistics and Group Differences

#### 3.1.1. Descriptive Statistics

To better understand the distribution patterns and potential differences in key variables across educational levels (master’s and doctoral students), we conducted descriptive statistical analyses and independent sample *t*-tests. Results are presented in Table 1.

The average age of master’s students was 24.21, while doctoral students had a significantly higher mean age of 27.77 (*t* = −38.21, *p* < 0.001), with a large effect size (Cohen’s *d* = 1.62). This substantial age gap suggests that developmental differences between the two groups should not be overlooked, thereby supporting the use of multi-group analyses in subsequent modeling. In terms of parental education background, the two groups displayed relatively similar distributions. However, doctoral students had a higher proportion of parents with a bachelor’s degree or above. Notably, 13.4% of doctoral students reported having a father with a graduate-level education, compared to only 8.4% among master’s students. This difference may reflect a greater accumulation of cultural capital in the families of doctoral students and implies the potential for intergenerational transmission of educational advantages.

Regarding self-reported family economic status, the majority of participants across both groups rated their family background as “moderate.” A slightly higher proportion of doctoral students reported “good” or “affluent” financial conditions, though the overall difference in economic background between the two groups was not statistically significant.

#### 3.1.2. Educational Stage Differences

Doctoral students scored significantly higher than master’s students on the quality of the supervisor–postgraduate relationship (*t* = −4.03, *p* < 0.001), with a small effect size (Cohen’s *d* = 0.18). While the effect is modest, it suggests that doctoral students may experience slightly more positive interactions with their advisors, potentially due to more frequent research collaboration. Doctoral students also reported significantly greater research pressure than master’s students (*t* = −2.86, *p* = 0.004, Cohen’s *d* = 0.13). This finding indicates that despite closer mentorship, doctoral students experience heightened academic research pressure. In terms of mental health as measured by the SCL-90, no significant difference was found between the two groups (*p* = 0.289), suggesting a similar level of overall psychological symptom burden across educational levels. However, doctoral students exhibited significantly higher scores on suicidal tendency compared to master’s students (*t* = −3.00, *p* = 0.003, Cohen’s *d* = 0.13). Although the effect size is small, the result is concerning and highlights an elevated risk of self-harm among doctoral students. Lastly, doctoral students showed significantly higher self-efficacy in research than master’s students (*t* = −5.85, *p* < 0.001, Cohen’s *d* = 0.26). This indicates greater confidence among doctoral students in their ability to manage research tasks, suggesting that educational level not only shapes stress experiences but may also moderate one’s perceived research competence.

### 3.2. Measurement Invariance

**Figure 1:**
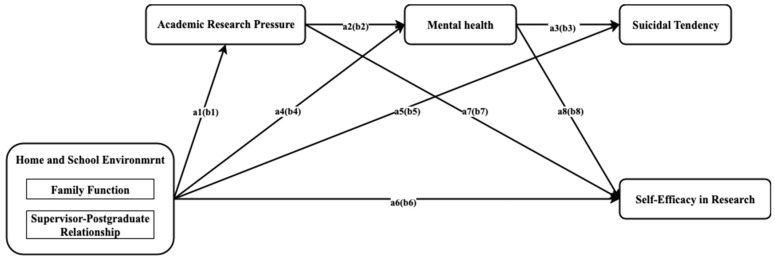
Path model diagram of structural equations at the master’s and doctoral stages. *Note*. The above figure shows the hypothetical path relationships among the various variables. Among them, paths a1 to a8 represent the master model, and paths b1 to b8 represent the doctoral model. The value represents the standardized path coefficient.

Although the chi-square test results were significant, the structural equation model (SEM) results (Figure 1) indicated that the hypothetical model had a relatively high degree of fit (*χ*^2^ = 44.071, *df* = 5, *p* < 0.001, *χ*^2^/*df* = 8.814, CFI = 0.992, RMSEA = 0.044). According to Kline (2015), model fit can be considered acceptable. The study provided partial evidence of structural invariance by testing the invariance of structural paths across educational stages (as shown in Table 2). Specifically, most of the equality constraints (a1, a2, a3, a6, a7 and a8) did not produce significant changes in model fitting (Δ*χ*^2^/*df* ≤ 2; ΔNFI, ΔIFI, ΔTLI ≤ 0.01), indicating that these paths are statistically equivalent between the two groups. However, for these two paths, the invariance assumption is not supported. When the constraint a4 = b4 is applied, the chi-square difference is significant (Δ*χ*^2^/*df* = 313.063, *p* < 0.001), and the change in the incremental fitting index also exceeds the recommended threshold, indicating that there are obvious structural differences between master’s and doctoral students on this path. In contrast, the equality constraint of a5 = b5 also produced statistically significant chi-square differences (Δ*χ*^2^/*df* = 8.261, *p* = 0.004), but the corresponding changes in the fitting index were insignificant (ΔCFI = 0.002). The structural model is basically stable among different groups, but some key approaches may vary depending on whether the respondents hold a master’s or doctoral degree.

### 3.3. Multi-Group Path Analysis

**Figure 2:**
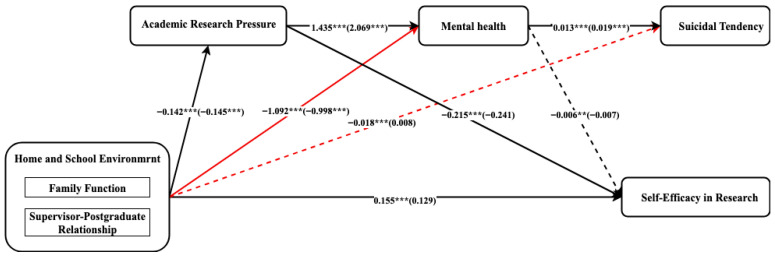
Results of the partially constrained models. *Note*. ** *p* < 0.01, *** *p* < 0.001; *β* is in parentheses for the doctoral students, while it is outside parentheses for the master; The dotted line indicates that the path coefficient at the doctoral stage is not significant; The red lines indicate that the structural coefficients of different educational stage groups are significantly different.

For the master’s stage, all the hypothetical structural paths were statistically significant, supporting the proposed model. In contrast, in the doctoral stage, neither of the two pathways, (a) the influence of home and school environment on suicidal tendency (*β* = 0.008, n.s.) and (b) the influence of mental health on self-efficacy in research (*β* = −0.241, n.s.), reached significance. This indicates that among doctoral students, the home and school environment have no direct impact on suicidal tendencies, and mental health has no significant influence on the study of self-efficacy in research. The comparison of nested models (Figure 2) further confirmed these differences. When equal constraints were imposed on the above two paths, the model fitting deteriorated significantly (home and school environment→suicidal tendency, Δ*χ*^2^ = 313.063, *p* < 0.001; home and school environment→mental health, Δ*χ*^2^ = 8.261, *p* = 0.004). For the remaining seven paths, the constraint equality between groups did not significantly reduce the model fitting (Δ*χ*^2^/*df* < 2, ΔCFI < 0.01), indicating invariance. Beyond path significance, the squared multiple correlations (*R*^2^) further demonstrated the model’s explanatory power. For master’s students, the model accounted for *R*^2^ = 0.281 of the variance in mental health, *R*^2^ = 0.519 in research self-efficacy, and *R*^2^ = 0.094 in suicidal tendency, suggesting substantial predictive strength. Among doctoral students, the corresponding values were *R*^2^ = 0.361, *R*^2^ = 0.514, and *R*^2^ = 0.081, respectively, indicating a moderate level of explanatory power and reduced predictive efficiency at the doctoral stage. Although *R*^2^ values were generally higher for master’s students, reflecting stronger mediation through self-efficacy, the model was selected primarily based on theoretical assumptions rather than empirical optimization.

These results provide evidence of partial structural invariance across educational stages. Although most structural mechanisms are shared between master’s and doctoral students, doctoral students show weaker or less significant associations in how the situational environment affects suicidal tendency and how mental health promotes self-efficacy in research. The differences among these specific groups suggest that as postgraduates progress academically, the determinants of mental health and efficacy may vary.

## 4. Discussion

Drawing on data from 3998 master’s and doctoral students, this study used multi-group structural equation modeling to examine how family function and supervisor–postgraduate relationships influence mental health, research self-efficacy, and suicidal ideation. The findings revealed clear stage-specific psychological mechanisms. Master’s students primarily benefited from a “positive incentive pathway”, in which family and mentor support enhanced self-efficacy and promoted psychological adaptation. Doctoral students, in contrast, followed a “risk accumulation pathway”, whereby family function indirectly contributed to suicidal ideation through increased academic research pressure and psychological symptoms. These patterns indicate that family–school support operates differently across educational stages and may reflect implicit biases in how psychological support is distributed.

### 4.1. Differential Impacts of Home and School Environment on Mental Health

Family and school environments significantly predicted psychological symptoms in both groups, supporting the stress-buffering hypothesis (Cohen & Wills, 1985) and prior findings in Chinese medical students (Shao et al., 2020). However, the underlying mechanisms varied. Master’s students, still in a transitional developmental stage, mainly relied on motivational support that strengthened self-efficacy and emotional well-being. Doctoral students faced heavier academic and social demands, including publication pressure, financial responsibilities, and familial expectations. For them, family and mentor support primarily reduced academic research pressure and buffered psychological risk. Supervisor–postgraduate relationships showed a dual nature: although doctoral students reported slightly better mentor quality, they also experienced higher stress and suicidal ideation, suggesting that hierarchical mentorship may transform support into stress. These findings align with Arnett’s (2000) stage-specific developmental model and Levecque et al.’s (2017) evidence linking doctoral workload to mental health risks.

### 4.2. Differences in the Chain of Psychological Mechanisms Shaped by Developmental Tasks

The findings indicate that postgraduates display distinct psychological mechanisms across developmental stages. For master’s students, a “positive incentive chain” was observed, in which family function enhanced research self-efficacy, promoting psychological adaptation and positive emotions (Li et al., 2025). At this transitional stage from student to professional, individuals are highly responsive to external support, and family and mentor resources readily translate into motivation and self-identity. Their larger cohort size and relatively diffuse social expectations further help distribute stress, facilitating positive reinforcement processes. In contrast, doctoral students tended to follow a “risk accumulation chain,” where family function indirectly affected suicidal tendency through academic research pressure and psychological symptoms (Bergvall et al., 2025). Multiple role conflicts, balancing research productivity with responsibilities related to marriage, finances, and social expectations, intensify the negative effects of stress and increase vulnerability to psychological symptoms. The supervisor–postgraduate relationship also plays a dual role: supportive mentorship alleviates pressure, whereas authoritarian supervision may heighten anxiety and role conflict (Mavrogalou-Foti et al., 2024; Li et al., 2025). Accordingly, mental health interventions should be tailored by stage, fostering self-efficacy and motivation among master’s students, and mitigating stress accumulation and role conflict among doctoral students.

### 4.3. “Responsibility Source” Role of Family–School Resources Under the Thick-Skin Bias

The “thick-skinned bias” may inadvertently exacerbate inequalities in resource allocation and amplify doctoral students’ stress. This bias creates a “Matthew effect,” where those perceived as resilient or high achieving receive more opportunities and support, while students facing silent struggles are systematically overlooked. Although doctoral students report stronger supervisor–postgraduate relationships than master’s students, they also experience higher stress, psychological symptoms, and suicidal tendencies, suggesting that perceived resilience masks underlying vulnerability (S. Liu et al., 2024). The bias may also suppress help-seeking behaviors, as students internalize social expectations to appear self-sufficient and avoid disclosing distress, rendering their struggles invisible to mentors and family members. Consequently, those lacking strong family support face a double disadvantage, fewer institutional resources and unrecognized psychological needs (Yuan et al., 2025). The nonsignificant direct pathway from home and school environments to suicidal ideation among doctoral students may reflect this disengagement from help-seeking. Consistent with cumulative advantage theory, students with early support maintain well-being, whereas those without it face escalating disadvantages. These findings underscore the developmental divergence between the two groups: while master’s students benefit primarily from motivational and emotional reinforcement, doctoral students require protective mechanisms that buffer cumulative and chronic stressors. Understanding these differentiated pathways provides a foundation for designing stage-specific psychological interventions and preventive strategies within graduate education.

### 4.4. Implications for Psychological Support and Policy Development

The findings of this study provide valuable insights for improving psychological support systems in graduate education. The stage-specific mechanisms identified suggest that mental health strategies should be differentiated between master’s and doctoral students. For master’s students, interventions should emphasize enhancing research self-efficacy, motivation, and emotional adjustment through constructive mentoring and supportive family engagement (Miao et al., 2025). For doctoral students, greater attention should be given to alleviating long-term academic research pressure, role conflicts, and potential emotional exhaustion through regular mental health assessments, supervisor communication training, and institutional monitoring mechanisms (Ueno et al., 2025). At a broader level, universities should strengthen cross-departmental collaboration among counselors, academic mentors, and administrative units to build a more inclusive mental health environment. Establishing preventive mechanisms and responsive support networks can help reduce hidden psychological risks and foster a culture of well-being throughout postgraduate education.

### 4.5. Research Limitations and Future Directions

The findings carry important clinical and practical implications for postgraduate mental health services. The clear distinction between the psychological mechanisms of master’s and doctoral students suggests that mental health interventions should be stage-specific and evidence-based. For master’s students, interventions should focus on strengthening research self-efficacy, emotional regulation, and adaptive motivation, as these protective factors form the core of the “positive incentive pathway.” Structured mentoring, skill-based workshops, and family involvement programs may enhance their sense of academic competence and belonging. For doctoral students, the prominence of the “risk accumulation pathway” highlights the need for preventive and therapeutic approaches targeting chronic stress, depressive symptoms, and suicidal ideation. Universities should integrate early identification and continuous monitoring systems, including regular mental health screening and counseling, into doctoral programs. Training supervisors in psychological literacy and empathetic communication can also mitigate stress stemming from hierarchical mentoring relationships. Furthermore, cross-disciplinary collaboration between psychologists, educators, and medical professionals is essential to establish a comprehensive mental health framework that provides both psychosocial and clinical support. By aligning institutional practices with clinical insights, universities can reduce the incidence of severe psychological outcomes and promote sustainable academic well-being.

## 5. Conclusions

The present study deepens understanding of how family–school support shapes the academic and psychological well-being of postgraduates. Using multi-group SEM, we found that the overall structural model remained stable across educational stages, yet two key mechanisms diverged. For master’s students, family and supervisor support enhanced research self-efficacy and indirectly improved mental health, forming a positive incentive pathway driven by motivation and emotional reinforcement. For doctoral students, however, the protective effects of contextual resources weakened: family and supervisor support no longer directly reduced suicidal tendency, and mental health showed a limited influence on self-efficacy. This pattern reflects a risk accumulation pathway, in which chronic academic pressure and role conflicts gradually erode the buffering function of support. In summary, master’s students benefit primarily from motivational and resource-based protection, whereas doctoral students are more vulnerable to stress accumulation and reduced psychological resilience. These findings highlight the need for stage-specific mental health interventions, enhancing self-efficacy and positive feedback for master’s students and reducing sustained stress and emotional exhaustion for doctoral students, to promote balanced and sustainable well-being in graduate education.

## Figures and Tables

**Table 1:** Descriptive statistics and difference tests for various variables between master’s students and doctoral students.

	Master’s Students (*n* = 3393)	Doctoral Students (*n* = 605)	*t*	*p*	Cohen’s *d*
Age (*M* ± *SD*)	24.21 ± 1.521	27.77 ± 2.841	–38.21 ***	<0.001	1.62
Gender					
Male	1636	380	-	-	-
Female	1757	225	-	-	-
Father’s Education					
Elementary or below	494	94	-	-	-
Junior high	1234	193	-	-	-
High school	946	155	-	-	-
Undergraduate	382	57	-	-	-
Graduate	286	81	-	-	-
Other	51	25	-	-	-
Mother’s Education					
Elementary or below	756	135	-	-	-
Junior high	1208	196	-	-	-
High school	888	146	-	-	-
Undergraduate	292	59	-	-	-
Graduate	214	54	-	-	-
Other	35	15	-	-	-
Family Economic Status					
Poor	437	58	-	-	-
Average	2337	396	-	-	-
Good	584	136	-	-	-
Wealthy	35	15	-	-	-
Core Research Variables					
Family Function	19.10 ± 5.12	19.24 ± 5.28	–0.6	0.551	0.03
Supervisor–Postgraduate Relationship	181.57 ± 18.21	184.82 ± 18.71	–4.03 ***	<0.001	0.18
Academic Research Pressure	10.75 ± 3.61	11.21 ± 3.74	–2.86 **	0.004	0.13
SCL-90 (Mental Health)	117.88 ± 32.08	116.38 ± 31.99	1.06	0.289	0.05
Suicidal Tendency	0.35 ± 1.88	0.60 ± 1.92	–3.00 **	0.003	0.13
Self-Efficacy in Research	11.82 ± 3.54	12.73 ± 3.56	–5.85 ***	<0.001	0.26

*Note.* *** *p* < 0.001, ** *p* < 0.01, etc., indicate levels of statistical significance. Cohen’s *d* indicates effect size.

**Table 2:** The model structure path invariance varies at different educational stages.

Restrictive Conditions	Δ*χ*^2^/*df*	*p*	ΔNFI	ΔIFI	ΔRFI	ΔTLI
a1 = b1	0.031	0.859	<0.001	<0.001	–0.004	–0.004
a2 = b2	1.585	0.208	<0.001	<0.001	–0.003	–0.003
a3 = b3	2.590	0.108	0.001	0.001	–0.002	–0.002
a4 = b4	313.063	<0.001	0.061	0.061	0.229	0.231
a5 = b5	8.261	0.004	0.002	0.002	0.002	0.002
a6 = b6	1.112	0.292	<0.001	<0.001	–0.003	–0.003
a7 = b7	0.294	0.588	<0.001	<0.001	–0.004	–0.004
a8 = b8	0.015	0.902	<0.001	<0.001	–0.004	–0.004

## Data Availability

The datasets presented in this article are not readily available because it used to support the findings of this study which are restricted by the the Ethics Committee, Department of Psychology, China University of Geosciences. In order to protect participant privacy, the data are prohibited from being made public. Requests to access the datasets should be directed to lilin@cug.edu.cn.

## References

[B1-ejihpe-15-00227] Aass L. K., Moen Ø. L., Skundberg-Kletthagen H., Lundqvist L. O., Schröder A. (2022). Family support and quality of community mental health care: Perspectives from families living with mental illness. Journal of Clinical Nursing.

[B2-ejihpe-15-00227] Acock A. C. (2005). Working with missing values. Journal of Marriage and Family.

[B3-ejihpe-15-00227] Arnett J. J. (2000). Emerging adulthood: A theory of development from the late teens through the twenties. American Psychologist.

[B4-ejihpe-15-00227] Bandura A. (1997). Self-efficacy: The exercise of control.

[B5-ejihpe-15-00227] Barzoki M. H., Toikko T. (2025). Family intimacy and depression: A comparative study among adolescents in Finland. Nordic Journal of Psychiatry.

[B6-ejihpe-15-00227] Bergvall J., Lundberg T., Eriksson M. (2025). The impact of PhD studies on mental health: A cross-sectional survey. Health Economics.

[B7-ejihpe-15-00227] Berryhill M. B., Smith J. (2021). College student chaotically-disengaged family functioning, depression, and anxiety: The indirect effects of positive family communication and self-compassion. Marriage & Family Review.

[B8-ejihpe-15-00227] Brown P., Jones A., Davies J. (2020). Shall I tell my mentor? Exploring the mentor-student relationship and its impact on students’ raising concerns on clinical placement. Journal of Clinical Nursing.

[B9-ejihpe-15-00227] Chang J., Wang S. W., Mancini C., McGrath-Mahrer B., Orama de Jesus S. (2020). The complexity of cultural mismatch in higher education: Norms affecting first-generation college students’ coping and help-seeking behaviors. Cultural Diversity & Ethnic Minority Psychology.

[B10-ejihpe-15-00227] Chen B., Wang W., Yang S. (2023). The impact of family functioning on depression in college students: A moderated mediation model. Journal of Affective Disorders.

[B11-ejihpe-15-00227] Cohen S., Wills T. A. (1985). Stress, social support, and the buffering hypothesis. Psychological Bulletin.

[B12-ejihpe-15-00227] DeWit D. J., DuBois D., Erdem G., Larose S., Lipman E. L. (2016). The role of program-supported mentoring relationships in promoting youth mental health, behavioral and developmental outcomes. Prevention Science.

[B13-ejihpe-15-00227] Dolson J. M., Deemer E. D. (2022). The relationship between perceived discrimination and school/work–family conflict among graduate student-parents. Journal of Career Development.

[B14-ejihpe-15-00227] Eccles J. S., Wigfield A. (2002). Motivational beliefs, values, and goals. Annual Review of Psychology.

[B15-ejihpe-15-00227] Evans T. M., Bira L., Beltran-Gastelum J., Weiss L. T., Vanderford N. (2017). Mental health crisis in graduate education: The data and intervention strategies. The FASEB Journal.

[B16-ejihpe-15-00227] Falk C. F. (2018). Are robust standard errors the best approach for interval estimation with nonnormal data in structural equation modeling?. Structural Equation Modeling: A Multidisciplinary Journal.

[B17-ejihpe-15-00227] Farsani M. A., Ghorbani B. D. (2024). Examining research motivation, self-efficacy, and anxiety in TEFL graduate students: A structural equation modelling approach. Research in Post-Compulsory Education.

[B18-ejihpe-15-00227] Garcia-Williams A. G., Moffitt L., Kaslow N. J. (2014). Mental health and suicidal behavior among graduate students. Academic Psychiatry.

[B19-ejihpe-15-00227] Graen G. B., Uhl-Bien M. (1995). Relationship-based approach to leadership: Development of leader-member exchange (LMX) theory of leadership over 25 years: Applying a multi-level multi-domain perspective. The Leadership Quarterly.

[B20-ejihpe-15-00227] Guidotti S., Fiduccia A., Pruneti C. (2024). Introversion, alexithymia, and hostility: A path analysis from personality to suicidal ideation among university students. Psychological Reports.

[B21-ejihpe-15-00227] Haim-Nachum S., Sopp M. R., Bonanno G. A., Levy-Gigi E. (2022). The lasting effects of early adversity and updating ability on the tendency to develop PTSD symptoms following exposure to trauma in adulthood. Cognitive Therapy and Research.

[B22-ejihpe-15-00227] Helson H. (1964). Adaptation-level theory.

[B23-ejihpe-15-00227] Kline R. B. (2015). Principles and practice of structural equation modeling.

[B24-ejihpe-15-00227] Layne C. M., Beck C. J., Rimmasch H., Southwick J. S., Moreno M. A., Hobfoll S. E. (2008). Promoting “resilient” posttraumatic adjustment in childhood and beyond: “Unpacking” life events, adjustment trajectories, resources, and interventions. Treating traumatized children.

[B25-ejihpe-15-00227] Le T. P., Hsu T., Raposa E. B. (2021). Effects of natural mentoring relationships on college students’ mental health: The role of emotion regulation. American Journal of Community Psychology.

[B26-ejihpe-15-00227] Lev E. L., Kolassa J., Bakken L. L. (2010). Faculty mentors’ and students’ perceptions of students’ research self-efficacy. Nurse Education Today.

[B27-ejihpe-15-00227] Levecque K., Anseel F., De Beuckelaer A., Van der Heyden J., Gisle L. (2017). Work organization and mental health problems in PhD students. Research Policy.

[B28-ejihpe-15-00227] Li X., Zhang Y., Wang L. (2025). PhD student–supervisor relationship and its impacts: A perspective of the interpersonal relationship model. Frontiers in Education.

[B29-ejihpe-15-00227] Liu C., Wang L., Qi R., Wang W., Jia S., Shang D., Shao Y., Yu M., Zhu X., Yan S., Chang Q., Zhao Y. (2019). Prevalence and associated factors of depression and anxiety among doctors: The mediating effect of mentoring relationships on the association between research self-efficacy and depression/anxiety. Psychology Research and Behavior Management.

[B30-ejihpe-15-00227] Liu S., Wang X., Teng H., Gao W., Wang J., Xu F., Song M., Yang L. (2024). Supervisor-postgraduate relationship and perceived stress: The mediating role of self-efficacy and the moderating role of psychological resilience. BMC Psychology.

[B31-ejihpe-15-00227] Mavrogalou-Foti E., Clarke A., Allen S. (2024). The supervisory relationship as a predictor of mental health outcomes in doctoral students in the United Kingdom. Frontiers in Psychology.

[B32-ejihpe-15-00227] Meda N., Pardini S., Slongo I., Bodini L., Zordan M. A., Rigobello P., Visioli F., Novara C. (2021). Students’ mental health problems before, during, and after COVID-19 lockdown in Italy. Journal of Psychiatric Research.

[B33-ejihpe-15-00227] Miao H., Zhao L., Wang Z. (2025). The effects of mentor support on Ed. D students’ research creativity: Mediating roles of research self-efficacy and learning engagement. Frontiers in Psychology.

[B34-ejihpe-15-00227] Miller P., Votruba-Drzal E., Coley R. L. (2019). Poverty and academic achievement across the urban to rural landscape: Associations with community resources and stressors. RSF: The Russell Sage Foundation Journal of the Social Sciences.

[B35-ejihpe-15-00227] Paglis L. L., Green S. G., Bauer T. N. (2006). Does adviser mentoring add value? A longitudinal study of mentoring and doctoral student outcomes. Research in Higher Education.

[B36-ejihpe-15-00227] Preacher K. J., Hayes A. F. (2004). SPSS and SAS procedures for estimating indirect effects in simple mediation models. Behavior Research Methods, *Instruments, & Computers*.

[B37-ejihpe-15-00227] Rose G. L. (2005). Group differences in graduate students’ concepts of the ideal mentor. Research in Higher Education.

[B38-ejihpe-15-00227] Shao R., He P., Ling B., Tan L., Xu L., Hou Y., Kong L., Yang Y. (2020). Prevalence of depression and anxiety and correlations between depression, anxiety, family functioning, social support and coping styles among Chinese medical students. BMC Psychology.

[B39-ejihpe-15-00227] Stubb J., Pyhältö K., Lonka K. (2011). Balancing between inspiration and exhaustion: PhD students’ experienced socio-psychological well-being. Studies in Continuing Education.

[B40-ejihpe-15-00227] Thomas Tobin C. S., Erving C. L., Barve A. (2021). Race and SES differences in psychosocial resources: Implications for social stress theory. Social Psychology Quarterly.

[B41-ejihpe-15-00227] Ueno A., Yu C., Curtis L., Dennis C. (2025). Job demands-resources theory extended: Stress, loneliness, and care responsibilities impacting UK doctoral students’ and academics’ mental health. Studies in Higher Education.

[B42-ejihpe-15-00227] Walton G. M., Cohen G. L. (2011). A brief social-belonging intervention improves academic and health outcomes of minority students. Science.

[B43-ejihpe-15-00227] Wang P., Xiong Z., Yang H. (2018). Relationship of mental health, social support, and coping styles among graduate students: Evidence from Chinese universities. Iranian Journal of Public Health.

[B44-ejihpe-15-00227] Wang Z. Y. (1984). The self-report symptom inventory, symptom check-list90, SCL-90. Shanghai Archives of Psychiatry.

[B45-ejihpe-15-00227] Wentzel K. R., Russell S., Baker S. (2016). Emotional support and expectations from parents, teachers, and peers predict adolescent competence at school. Journal of Educational Psychology.

[B46-ejihpe-15-00227] Xu R. H., Zhou L. M., Wang D. (2021). The relationship between decisional regret and well-being in patients with and without depressive disorders: Mediating role of shared decision-making. Frontiers in Psychiatry.

[B47-ejihpe-15-00227] Yang L., Hou X. Q., Liu H. L. (2021). Development and validation of suicidal behavior screening questionnaire. Chinese Journal of Clinical Psychology.

[B48-ejihpe-15-00227] Yang Q., Hu Y. Q., Zeng Z. H., Liu S. J., Wu T., Zhang G. H. (2022). The relationship of family functioning and suicidal ideation among adolescents: The mediating role of defeat and the moderating role of meaning in life. International Journal of Environmental Research and Public Health.

[B49-ejihpe-15-00227] Yuan W., Ning J., Huo M., Feng Y. (2025). The relationship between social support and psychological crisis vulnerability among family impoverished undergraduates: The intermediary role of psychological resilience. Frontiers in Public Health.

[B50-ejihpe-15-00227] Zhang J., Goodson P. (2011). Predictors of international students’ psychosocial adjustment to life in the United States: A systematic review. International Journal of Intercultural Relations.

[B51-ejihpe-15-00227] Zhang Y. J., Liao J. Q., Zhao J. (2013). The impact of research stress on the academic misconduct of PhD candidates. Science Research Management.

[B52-ejihpe-15-00227] Zhou F. (2009). A study on the effect of perceived team support, research self-efficacy on innovation performance in university research teams. Master’s thesis.

[B53-ejihpe-15-00227] Zhou H., Long L. R. (2004). Statistical remedies for common method biases. Advances in Psychological Science.

[B54-ejihpe-15-00227] Zou H., Li X. W., Zhang W. J. (2010). The characteristics of adolescents’ family relationship and its effect on adolescents’ social adjustment. Psychological Science.

